# Supplementary material for: Robust prognostic prediction model developed with integrated biological markers for acute myocardial infarction
Source: PLoS One. 2022 Nov 3;17(11):e0277260. doi: 10.1371/journal.pone.0277260 (PMC9632913; doi:10.1371/journal.pone.0277260)
Supplement: S1 Table — (DOCX) [file pone.0277260.s001.docx]

**S1 Table. Characteristics of patients in training and test subsets**

| **Characteristics** | **Training (n = 2,042)** | **Test (n = 511)** |
| --- | --- | --- |
| Prognosis dead, n (%) | 158 (7.7) | 37 (7.2) |
| Age, median (IQR) | 70 (62–79) | 69 (61–78) |
| Gender female, n (%) | 553 (27.5) | 130 (25.7) |
| BMI, median (IQR) | 23.0 (20.7–25.4) | 22.8 (20.8–25.3) |
| Hypertension, n (%) | 1258 (63.5) | 287 (58.0) |
| Diabetes, n (%) | 620 (31.3) | 149 (30.1) |
| Dyslipidaemia, n (%) | 874 (44.1) | 201 (40.6) |
| Smoking, n (%) | 779 (39.3) | 220 (44.5) |
| Family history, n (%) | 137 (6.9) | 34 (6.8) |
| MI history, n (%) | 172 (8.6) | 59 (11.8) |
| CVD history, n (%) | 426 (21.4) | 111 (22.2) |
| HD, n (%) | 44 (2.1) | 11 (2.1) |
| Killip, n (%) |  |  |
| 1 | 1305 (69.4) | 324 (68.7) |
| 2 | 256 (13.6) | 77 (16.3) |
| 3 | 111 (5.9) | 24 (5.0) |
| 4 | 207 (11.0) | 46 (9.7) |
| BPs, median (IQR) | 131 (110–153) | 132 (112–152) |
| HR, median (IQR) | 74 (60–89) | 72 (60–88) |
| WBC, median (IQR) | 9490 (7670–11930) | 9710 (7500–12000) |
| Hb, median (IQR) | 14.0 (12.6–15.3) | 14.1 (12.5–15.3) |
| BS, median (IQR) | 155 (128–207) | 158 (129–202) |
| max CPK, median (IQR) | 1916 (875–3582) | 1703 (753–3302) |
| Cr, median (IQR) | 0.8 (0.7–1.0) | 0.8 (0.7–1.1) |
| CRP, median (IQR) | 0.1 (0.07–0.65) | 0.1 (0.08–0.5) |
| TIMI pre, n (%) |  |  |
| 0 | 1212 (59.4) | 301 (59.4) |
| 1 | 213 (10.4) | 65 (12.8) |
| 2 | 365 (17.9) | 68 (13.4) |
| 3 | 249 (12.2) | 72 (14.2) |
| TIMI post, n (%) |  |  |
| 0 | 39 (1.9) | 8 (1.5) |
| 1 | 24 (1.1) | 8 (1.5) |
| 2 | 112 (5.5) | 31 (6.1) |
| 3 | 1848 (91.3) | 454 (90.6) |
| Stent, n (%) | 1781 (88.2) | 441 (88.3) |
| Thrombus aspiration, n (%) | 1369 (67.8) | 342 (68.5) |
| Part of MI, n (%) |  |  |
| Anterior | 1040 (51.1) | 251 (49.7) |
| Postero–inferior | 844 (41.5) | 212 (41.9) |
| Lateral | 149 (7.3) | 42 (8.3) |
| Culprit vessel, n (%) |  |  |
| RCA | 790 (39.0) | 196 (38.9) |
| LAD | 978 (48.2) | 245 (48.7) |
| LMT | 53 (2.6) | 8 (1.5) |
| LCX | 204 (10.0) | 54 (10.7) |
| Numbers of lesion, n (%) |  |  |
| 1 | 1221 (60.8) | 302 (60.8) |
| 2 | 528 (26.2) | 126 (25.4) |
| 3 | 259 (12.8) | 68 (13.7) |
| Season, n (%) |  |  |
| Spring | 538 (26.3) | 121 (23.6) |
| Summer | 457 (22.3) | 120 (23.4) |
| Autumn | 475 (23.2) | 131 (25.6) |
| Winter | 572 (28.0) | 139 (27.2) |
| Onset time, n (%) |  |  |
| Diurnal | 985 (55.9) | 262 (59.0) |
| Nocturnal | 777 (44.0) | 182 (40.9) |
| Door to balloon time, n (%) |  |  |
| ≦ 1.5 h | 69 (3.5) | 15 (3.0) |
| 1.5 h < | 1878 (96.4) | 474 (96.9) |
| Onset to door time, n (%) |  |  |
| ≦ 24 h | 1745 (87.2) | 437 (87.5) |
| 24 h < | 254 (12.7) | 62 (12.4) |
| Transfer, n (%) |  |  |
| Ambulance | 955 (48.4) | 250 (50.4) |
| Walk–in | 511 (25.9) | 137 (27.6) |
| In–hospital | 67 (3.4) | 17 (3.4) |
| From other hospital | 437 (22.1) | 92 (18.5) |
| Grace Score, median (IQR) | 161 (140–186) | 159 (139–186) |
| TIMI risk index, median (IQR) | 26.7 (18.8–37.3) | 25.5 (17.7–36.1) |

IQR, interquartile range; BMI, body mass index; MI, myocardial infarction; CVD, cardiovascular disease; HD, hemodialysis; BPs, systolic blood pressure; HR, heart rate; WBC, white blood cells; Hb, hemoglobin; BS, blood sugar; CPK, creatine phosphokinase; Cr, creatinine; CRP, c-reactive protein; TIMI, thrombolysis in myocardial infarction; RCA, right coronary artery; LAD, left anterior descending artery; LMT, left main trunk; LCX, left circumflex.
