# Supplementary figures and images for: Robust prognostic prediction model developed with integrated biological markers for acute myocardial infarction
Source: PLoS One. 2022 Nov 3;17(11):e0277260. doi: 10.1371/journal.pone.0277260 (PMC9632913; doi:10.1371/journal.pone.0277260)

**
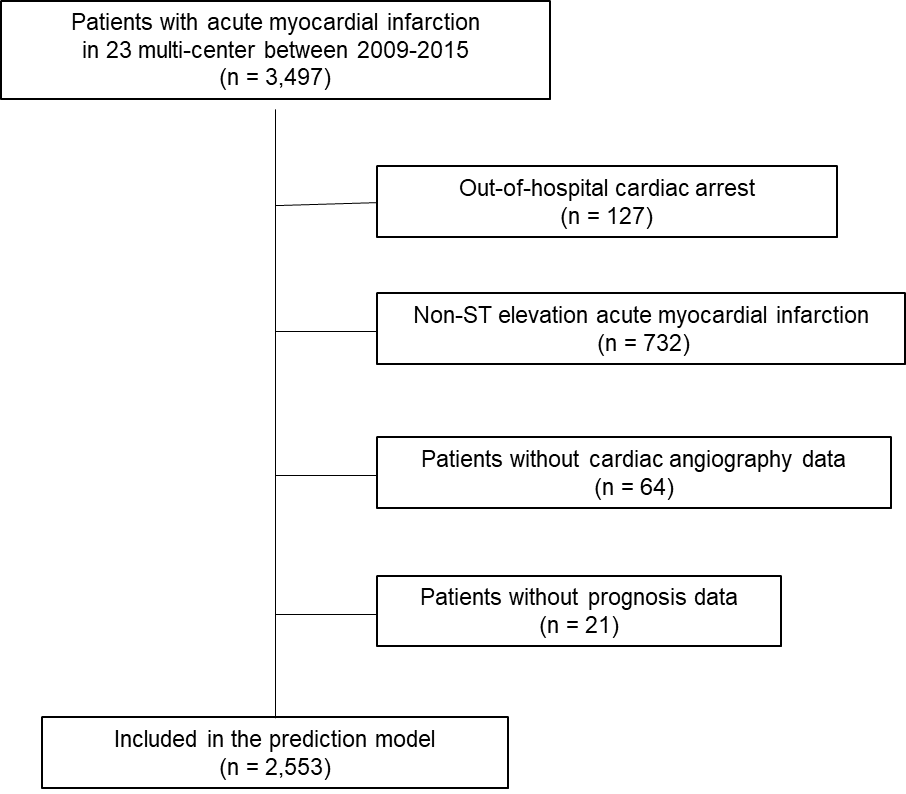
**

**S1 Fig. Flow diagram of sample collection and filtering process**

Supplement: S1 Fig — (DOCX) [file pone.0277260.s002.docx]
