# Supplementary material for: Robust prognostic prediction model developed with integrated biological markers for acute myocardial infarction
Source: PLoS One. 2022 Nov 3;17(11):e0277260. doi: 10.1371/journal.pone.0277260 (PMC9632913; doi:10.1371/journal.pone.0277260)
Supplement: S2 Fig — Online application tool of KOTOMI model to predict in-hospital mortality risk in patients with ST-elevation acute myocardial infarction (STEMI) was developed in Heroku web application platform according to the programming code of the model. An application programming interface (API) that returns the prediction results was tested to show the correct behavior for a subset of records in the dataset. (DOCX) [file pone.0277260.s003.docx]

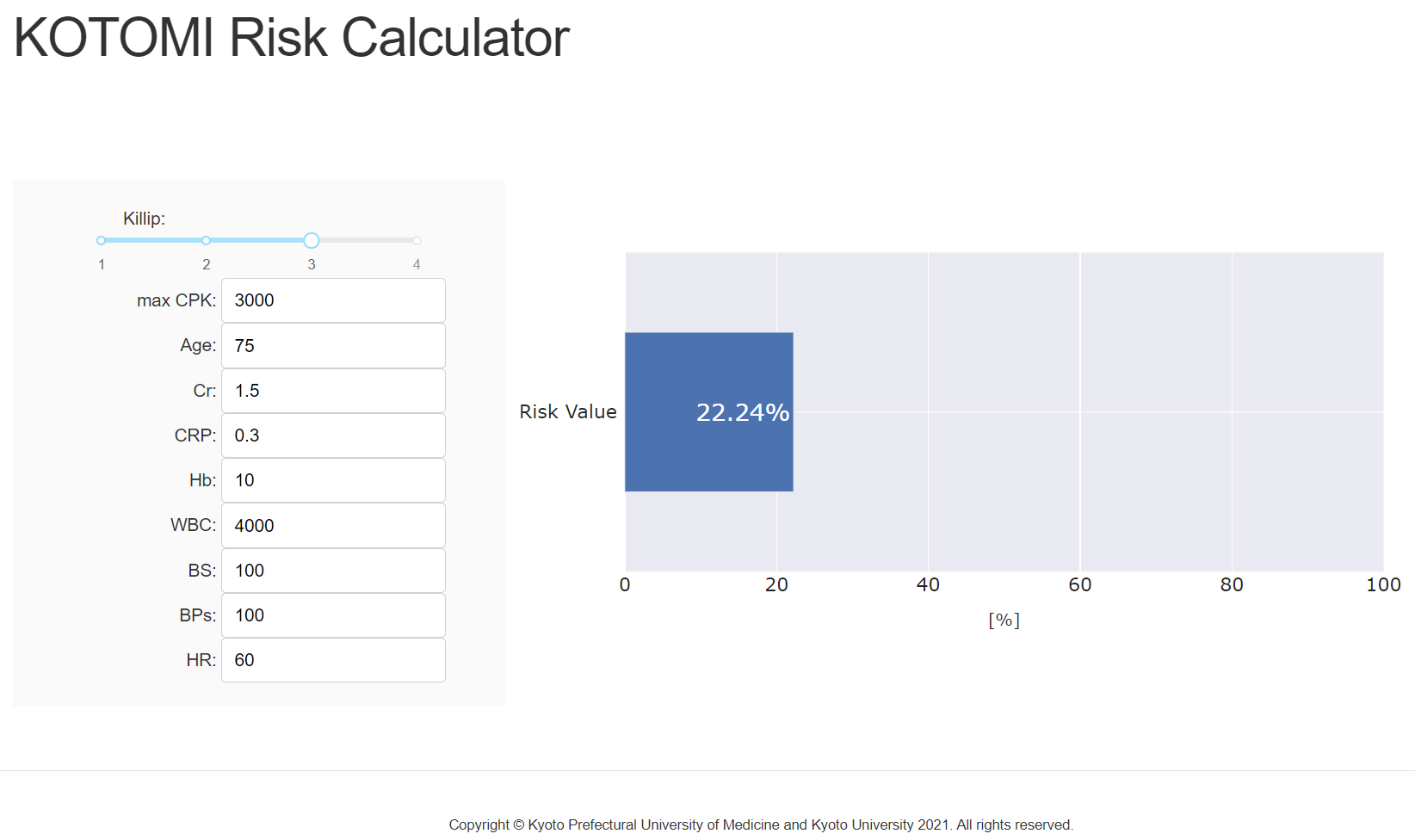


**S2 Fig. KOTOMI Risk Calculator for in-hospital mortality risk in patients with acute myocardial infarction.**

Online application tool of KOTOMI model to predict in-hospital mortality risk in patients with ST-elevation acute myocardial infarction (STEMI) was developed in Heroku web application platform according to the programming code of the model. An application programming interface (API) that returns the prediction results was tested to show the correct behavior for a subset of records in the dataset.
