# Supplementary material for: Robust prognostic prediction model developed with integrated biological markers for acute myocardial infarction
Source: PLoS One. 2022 Nov 3;17(11):e0277260. doi: 10.1371/journal.pone.0277260 (PMC9632913; doi:10.1371/journal.pone.0277260)
Supplement: S3 Fig — AUPRC indicates area under precision-recall curve. (DOCX) [file pone.0277260.s004.docx]

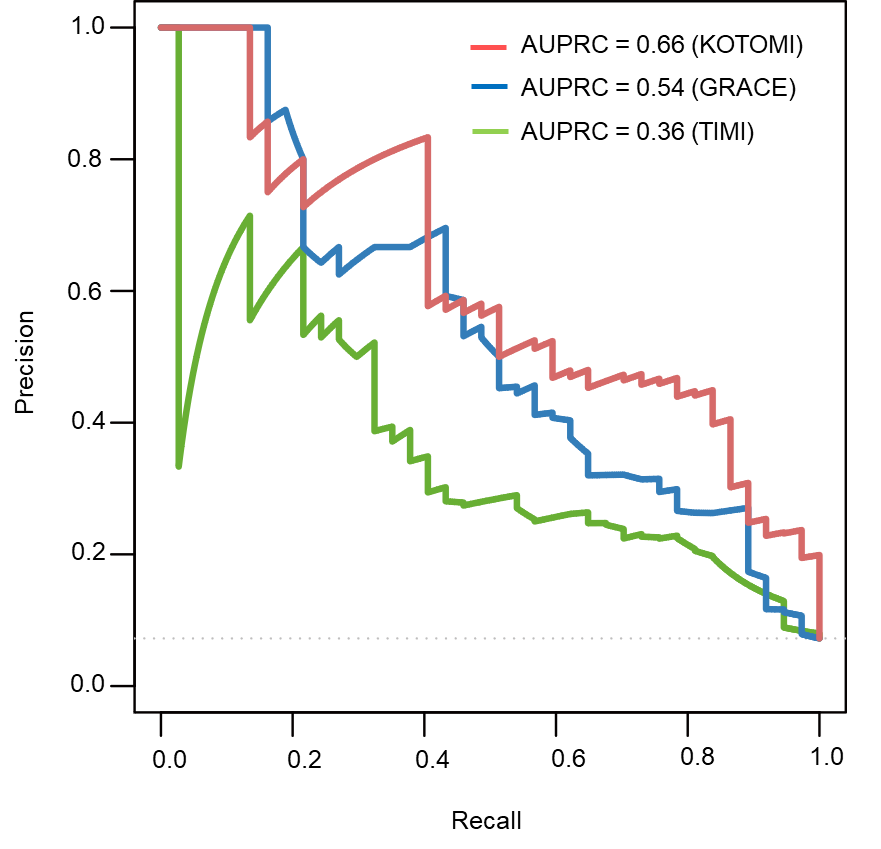


**S3 Fig. Model performance illustrated by precision-recall curve for test subset in KOTOMI, GRACE Score, and TIMI risk index.**

AUPRC indicates area under precision-recall curve.
