# Supplementary material for: Robust prognostic prediction model developed with integrated biological markers for acute myocardial infarction
Source: PLoS One. 2022 Nov 3;17(11):e0277260. doi: 10.1371/journal.pone.0277260 (PMC9632913; doi:10.1371/journal.pone.0277260)
Supplement: S4 Fig — Calibration plots were depicted for original model (left panel) and model calibrated by Isotonic Regression (right panel) using training data (A) and test data (B). Samples were divided into ten bins. (DOCX) [file pone.0277260.s005.docx]

**
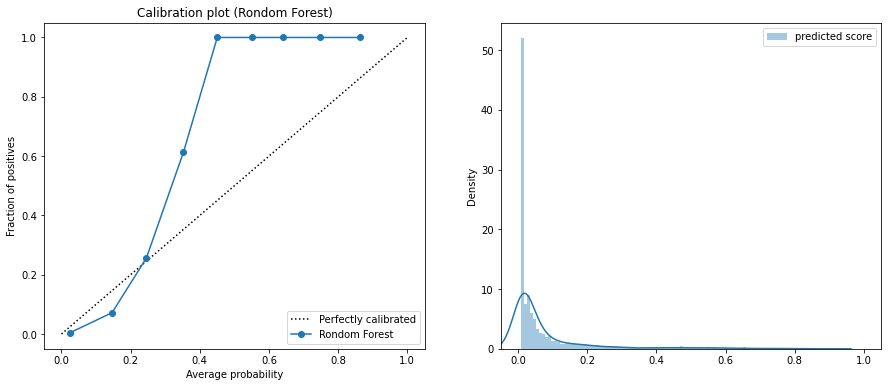

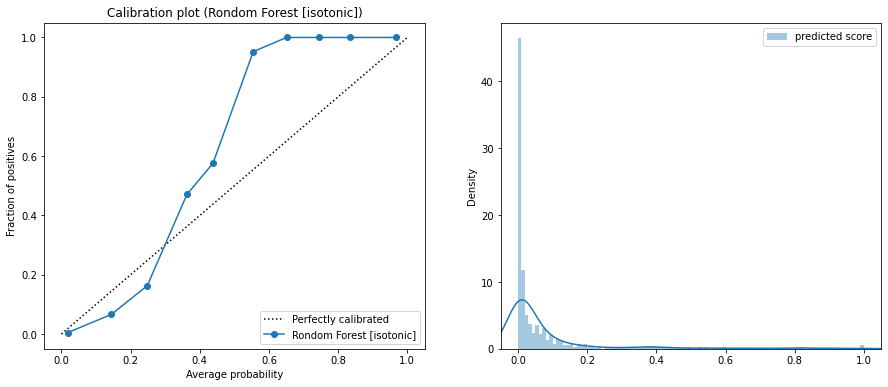
A**


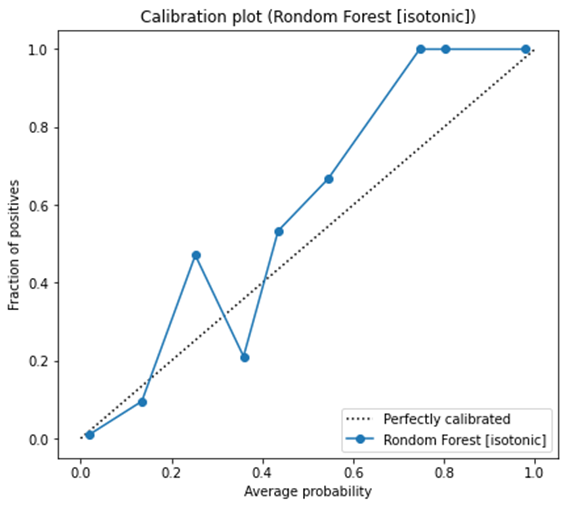
**B
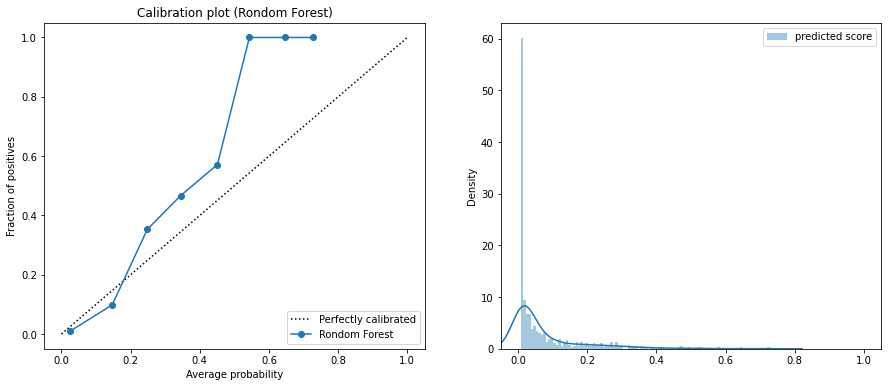
**

**S4 Fig. Model calibration.**

Calibration plots were depicted for original model (left panel) and model calibrated by Isotonic Regression (right panel) for training data (A) and test data (B). Samples were divided into ten bins.
