# Supplementary material for: Robust prognostic prediction model developed with integrated biological markers for acute myocardial infarction
Source: PLoS One. 2022 Nov 3;17(11):e0277260. doi: 10.1371/journal.pone.0277260 (PMC9632913; doi:10.1371/journal.pone.0277260)
Supplement: Attachment — Submitted filename: Peer review do artigo do PLOS One.docx [file pone.0277260.s007.docx]

**Peer eview: Robust prognostic prediction model developed with integrated biological markers for acute myocardial infarction**

The authors present a very interesting contemporary approach to assess in-hospital mortality of japanese patients admitted to the emergency ward with a diagnosis of S-T elevation acute myocardial infarction. They developed a machine-learning based tool that tested several sets of variables combined from a training cohort to find out the best combination of datasets associated with the variable of interest and tested it in a validation subset corresponding to 20% of patients from the entire cohort. In addition, the same cohort was assessed by the TIMI and the GRACE risk score, for the purpose of comparing the discriminatory power of the three methods. I was favorably impressed by the absence of any influence determined by anatomical variables or technical details associated with cateter-based interventions. The clinical, anatomic and laboratory baseline characteristics between the training and testing subsets were well balanced (table S1) and the precision-recall curve, in my opinion, was a wise choice, because the main variable of interest was the proportion of deaths (event) which, arguably, is expected to be much less frequent than the proportion of survivors (No event). Thus, unless I am mistaken, since I'm not a statistician, this method can provide a satisfactory way to **calibrate** the model. But I would thank the authors if they can provide me their thoughts about the need for calibration of the model. Another positive feature of this model is its limitation to predict a clearly identified outcome, i.e., death, and in a setting where the influence of unmeasured or uncontrolled factors is minimized, as opposed to what might be the case of death ocurring in an outpatient setting. The authors deserve congratulations for this elegant study that will positively contribute to improve the risk stratification of S-T elevation AMI patients in Japan.

I am not an expert in artificial intelligence and machine-learning methods, so I will limit my comments regarding this specific topic to some basic concepts. Having said this, here are some aspects that I considered worthy of comments:

1) I wonder why troponin measurements were not mentioned in their investigation. Could the authors provide an explanation for having chosen CPK instead of troponin?
2) Intuitively, it seems to me that the sample size for the model construction was large enough to predict one single and readily available outcome. Anyway, I wish to ask if the authors use any method or formula for sample size calculation.
3) Did the authors make sure that treatment strategies were similar for all centers enrolled, as well as if PCI volume and pharmacologic approaches were tantamount among the participating centers?
4) Since data collection and training were based upon electronic health records and were by definition retrospective, secular trends that potentially change the nature of care received by the patients may not be captured by the final model. I realize that the authors limited the data retrieval to a relatively narrow time range: between 2009 and 2015. However I suggest that they discuss whether this aspect might be regarded as a limitation or if they can discard it as a potential influence over the applicability of the model to the japanese population.
5) Did the authors consider that some of the inputs may have their influence decayed over time? I consider that one of the strenghts of this study was exactly the limited number of variables selected for input, namely 32. Anyway, I suggest that they take this detail into account and mention it at the discussion section.
6) Structured EHR labels and administrative codes may differ among institutions and influence the model accuracy. Did the authors control for these potential biases?
7) Regarding figure 3, which depicts the Kotomi risk calculator, I would suggest that the authors remove it from the main manuscript and move it to the supplementary supporting information. The web-based application is dynamic and the probability of the outcome, changes, as data are inputed. Upon reading the manuscript, I saw the URL and was immediately interested in watching the model performance. As opposed to the living experience itself, a still image cannot capture its dynamic nature. In summary, the reader’s curiosity will take him or her to the calculating tool. The still picture is supplementary, IMHO.
8) In the PLOS One recommendations f or submissions webpage, the section related to softwares and online tools publishes the following advice: “-For software, databases, and online tools, the long-term utility should also be discussed, as relevant. This discussion may include maintenance, the potential for future growth, and the stability of the hosting, as applicable. Inasmuch standards of care change overtime, can the authors estimate how they intend to update the model? So, in accordance to these recommendations, I suggest that the authors provide some comments over these aspects.
9) In the original TIMI publication, over 94% of patients were white, while in the original GRACE publication, there is no tabulation of ethnicity (Subsequent publications from the same registry ended up by extending the registry to asian populations). I wold ask the authors if at least part of the superior performance of the Kotomi model as compared to GRACE and TIMI, might also be influenced by a very different ethnic background. The authors state clearly that the set of variables comprising the model and its final presentation cannot be extrapolated to other ethnicities. However, by comparing it with GRACE and TIMI scores, the same limitations may at least in part account for the diferences in performance, regardless of the widespread use of PCI and other modern supportive approaches. Could the authors elaborate over this comment?
