# Supplementary material for: Robust prognostic prediction model developed with integrated biological markers for acute myocardial infarction
Source: PLoS One. 2022 Nov 3;17(11):e0277260. doi: 10.1371/journal.pone.0277260 (PMC9632913; doi:10.1371/journal.pone.0277260)
Supplement: Attachment — Submitted filename: Response to Reviewers.docx [file pone.0277260.s008.docx]

**Response to Reviewers’ comments**

**Academic editor:**

Please provide more details about the calibration of the generated model.
The lack of external validation may limit the model's applicability. Please comment and perhaps include it as a potential limitation.

**RESPONSE: Thank you for raising the important points.** **In the present study, we refrained from defining the prediction probability as ‘the mortality rate’. We performed the model calibration and edited the result of the manuscript with a new supporting figure (S4 Fig) as follows:**

**“Calibration plots showed that high fractions of positives tend to have lower probability than perfect calibration. In the present study, we used the original model for risk calculation (S4 Fig).”**

**A**

**B**

**S4 Fig. Model calibration.**

Calibration plots were depicted for original model (left panel) and model calibrated by Isotonic Regression (right panel) using training data (A) and test data (B). Samples were divided into ten bins.

**Also, we amended the discussion of the manuscript as follows:**

**“To enhance the model’s applicability, external validation should be performed.”**

**Reviewer #1**:

In this manuscript, the authors presented the results of a machine learning tool dedicated to the detection of STEMI patients at greater risk of death. The algorithm considered the AMI-Kyoto Multi-Center Risk Study, a multicenter national cohort of acute myocardial infarction held in Japan, which enrolled consecutive acute myocardial infarction patients who were admitted at 23 different hospitals across their country. Based on a well-designed random forest classifier model, the authors found an overwhelming accuracy for in-hospital mortality risk prediction, which overcame the accuracy of traditional risk scores, namely GRACE and TIMI. I recommend this paper should be considered for publication, as it may contribute to the application of ML tools dedicated to risk stratification of STEMI.

**RESPONSE: Thank you for gracious comments. We believe that the prognostic prediction model can be applied to risk stratification of STEMI as the reviewer mentioned.**

**Reviewer #2**:

The authors present a very interesting contemporary approach to assess in-hospital mortality of japanese patients admitted to the emergency ward with a diagnosis of S-T elevation acute myocardial infarction. They developed a machine-learning based tool that tested several sets of variables combined from a training cohort to find out the best combination of datasets associated with the variable of interest and tested it in a validation subset corresponding to 20% of patients from the entire cohort. In addition, the same cohort was assessed by the TIMI and the GRACE risk score, for the purpose of comparing the discriminatory power of the three methods. I was favorably impressed by the absence of any influence determined by anatomical variables or technical details associated with cateter-based interventions. The clinical, anatomic and laboratory baseline characteristics between the training and testing subsets were well balanced (table S1) and the precision-recall curve, in my opinion, was a wise choice, because the main variable of interest was the proportion of deaths (event) which, arguably, is expected to be much less frequent than the proportion of survivors (No event). Thus, unless I am mistaken, since I'm not a statistician, this method can provide a satisfactory way to calibrate the model. But I would thank the authors if they can provide me their thoughts about the need for calibration of the model. Another positive feature of this model is its limitation to predict a clearly identified outcome, i.e., death, and in a setting where the influence of unmeasured or uncontrolled factors is minimized, as opposed to what might be the case of death ocurring in an outpatient setting. The authors deserve congratulations for this elegant study that will positively contribute to improve the risk stratification of S-T elevation AMI patients in Japan.

I am not an expert in artificial intelligence and machine-learning methods, so I will limit my comments regarding this specific topic to some basic concepts. Having said this, here are some aspects that I considered worthy of comments:

**RESPONSE: Thank you for the kindly positive comments for our manuscript. In the present study, we refrained from defining the prediction probability as ‘the mortality rate’. In terms of the need of calibration, we performed the model calibration and edited the result of the manuscript with a new supporting figure (S4 Fig) as described in the response to the academic editor.**

1) I wonder why troponin measurements were not mentioned in their investigation. Could the authors provide an explanation for having chosen CPK instead of troponin?

**RESPONSE: Thank you for the important comment. We need to include the absence of troponin as a study limitation. Currently, troponin is a standard biomarker for early detection of myocardial infarction. However, the AMI-Kyoto Multi-Center Risk Study have continued to using only creatine kinase as a variable instead of troponin. As the reviewer pointed out, troponin is also associated with the severity of acute cardiac syndrome including STEMI. Therefore, we stated the absence of troponin and other important biomarkers as a study limitation in the discussion of the manuscript as follows:**

**“Troponin, which is a standard biomarker for diagnosis of STEMI and associated with its severity, CK-MB, BNP and NT-proBNP were not measured in the study.”**

2) Intuitively, it seems to me that the sample size for the model construction was large enough to predict one single and readily available outcome. Anyway, I wish to ask if the authors use any method or formula for sample size calculation.

**RESPONSE: Thank you for the constructive comments. As the reviewer indicated, power calculation is generally performed to estimate sample size to produce the statistical significance before the study starts. However, the present study is designed for machine learning prediction. In that case, power calculation is not necessary because statistical significance is not evaluated and model accuracy is the most important metrics. Accordingly, we edited the method of the manuscript as follows:**

**“****Power calculation was not performed, given that machine learning-based prediction does not evaluate statistical significance.”**

3) Did the authors make sure that treatment strategies were similar for all centers enrolled, as well as if PCI volume and pharmacologic approaches were tantamount among the participating centers?

**RESPONSE: Thank you for letting us notice the important points. PCI volume somewhat vary among the hospitals but the indication for PCI and pharmacological approaches are similar because all the hospitals are registered as training facility of Japanese Circulation Society and conduct the guideline-based standard management. We edited the method of the manuscript as follows:**

**“****Indication for PCI and pharmacological approaches are similar because all the hospitals are registered as training facility of Japanese Circulation Society and conduct the guideline-based standard management.”**

4) Since data collection and training were based upon electronic health records and were by definition retrospective, secular trends that potentially change the nature of care received by the patients may not be captured by the final model. I realize that the authors limited the data retrieval to a relatively narrow time range: between 2009 and 2015. However I suggest that they discuss whether this aspect might be regarded as a limitation or if they can discard it as a potential influence over the applicability of the model to the japanese population.

**RESPONSE: Thank you for the constructive suggestion. Actually, we could not correct the more current data because the patient enrollment was paused between 2016 to 2019 due to internal reason. The current therapeutic strategy including percutaneous coronary intervention for acute myocardial infarction has been already established, and not so changed today. According to the reviewer’s suggestion, we mention the narrow time range as the limitation of the manuscript as follows:**

**“The model was created using the data between January 2009 to December 2015. Validation for current data will be necessary for further study.”**

5) Did the authors consider that some of the inputs may have their influence decayed over time? I consider that one of the strenghts of this study was exactly the limited number of variables selected for input, namely 32. Anyway, I suggest that they take this detail into account and mention it at the discussion section.

**RESPONSE: We appreciate the reviewer for this suggestion. We agree that the selected inputs are not eternal variables. Indeed, biomarkers and therapeutic strategy for acute myocardial infarction will be upgraded timely. Thus, we include it in the discussion of the manuscript as follows:**

**“The selected features may need to be updated in the future using the contemporary data according to the progress of the standard management for acute myocardial infarction, followed by the update of its application tool.”**

6) Structured EHR labels and administrative codes may differ among institutions and influence the model accuracy. Did the authors control for these potential biases?

**RESPONSE: In this study, the data were collected manually by physicians. Therefore, data handling with EHR labels and administrative codes did not influence the study results. We appreciate the reviewer for the comments. We’ll consider the potential bias for further study.**

7) Regarding figure 3, which depicts the Kotomi risk calculator, I would suggest that the authors remove it from the main manuscript and move it to the supplementary supporting information. The web-based application is dynamic and the probability of the outcome, changes, as data are inputed. Upon reading the manuscript, I saw the URL and was immediately interested in watching the model performance. As opposed to the living experience itself, a still image cannot capture its dynamic nature. In summary, the reader’s curiosity will take him or her to the calculating tool. The still picture is supplementary, IMHO.

**RESPONSE: Thank you for the constructive comments. We recomposed the figures by translocating figure 3 to supplementary figure (S2 Fig).**

8) In the PLOS One recommendations f or submissions webpage, the section related to softwares and online tools publishes the following advice: “-For software, databases, and online tools, the long-term utility should also be discussed, as relevant. This discussion may include maintenance, the potential for future growth, and the stability of the hosting, as applicable. Inasmuch standards of care change overtime, can the authors estimate how they intend to update the model? So, in accordance to these recommendations, I suggest that the authors provide some comments over these aspects.

**RESPONSE: Thank you for a useful suggestion. We edited the discussion of the manuscript as with the response to comment 5):**

**“****The selected features may need to be updated in the future using the contemporary data according to the progress of the standard management for acute myocardial infarction, followed by the update of its application tool.”**

9) In the original TIMI publication, over 94% of patients were white, while in the original GRACE publication, there is no tabulation of ethnicity (Subsequent publications from the same registry ended up by extending the registry to asian populations). I wold ask the authors if at least part of the superior performance of the Kotomi model as compared to GRACE and TIMI, might also be influenced by a very different ethnic background. The authors state clearly that the set of variables comprising the model and its final presentation cannot be extrapolated to other ethnicities. However, by comparing it with GRACE and TIMI scores, the same limitations may at least in part account for the diferences in performance, regardless of the widespread use of PCI and other modern supportive approaches. Could the authors elaborate over this comment?

**RESPONSE: We appreciate the reviewer for all the comments. We agree that the racial difference should be considered as the study limitation for the performance comparison. Therefore, we edited the discussion of the manuscript as follows:**

**“Therefore, the model performance comparing with TIMI risk index and GRACE will need to be evaluated in larger cohorts of various races.”**

**Additional Reviewer:**

First, I would like to congrats the research and its design. It is a multicenter study with interesting sample size. However, I need some information

- Is there any data about image methods, such as an echocardiogram or cardiac resonance?

**RESPONSE: Thank you for the important comments. The imaging data was not corrected in the study. As described in the manuscript, left ventricular ejection fraction (LVEF) was removed from the patient’s characteristics because of the large number of missing values. Beside on it, we mentioned the lack of the imaging data in the discussion of the manuscript as follows:**

**“****Imaging data, such as echocardiogram or cardiac magnetic resonance, may be used as additional features for the prediction.”**

- Why did you choose CPK instead of troponin and CK-MB? I also would like to know about BNP or NT PRO BNP

**RESPONSE: We really agree with the reviewer’s comments. The study did not correct other biomarkers, such as** **troponin, CK-MB, BNP, and NT-proBNP. Therefore, we edited the discussion of the manuscript as follows:**

**“****Troponin, which is a standard biomarker for diagnosis of STEMI and associated with its severity, CK-MB, BNP and NT-proBNP were not measured in the study.”**

- About the calculator, did you perform API in order to protect the code?

**RESPONSE: We thank the reviewer for the question. The model development and validation source code can be reproduced according to the methods described in the manuscript. We have created a web tool (KOTOMI Risk Calculator) to make it possible for everyone to use the prediction model. An application programming interface (API) was built into the tool and tested to exhibit correct behavior for a subset of records in the dataset. Then, we added the following description to the figure legend of S2 Fig as follows:**

**“An application programming interface (API) that returns the prediction results was tested to show the correct behavior for a subset of records in the dataset.”**

- About the training and training subset, please clarify it:

What percentage of patients with the outcome of death were selected in each group?

**RESPONSE: Thank you for the comment. We showed the percentage of patients dead in S1 Table as below:**

| **Characteristics** | **Training (n = 2,042)** | **Test (n = 511)** |
| --- | --- | --- |
| Prognosis dead, n (%) | 158 (7.7) | 37 (7.2) |
